# Notes and News

**Published:** 1887-01-29

**Authors:** 


### Notes and News.

Collected and Communicated.

The Princess of Wales has added her name to the list of patronesses of Sir Patrick Dun's Hospital, Dublin.

Her Royal Highness the Princess Christian has kindly promised her patronage of the representation of "The Story of St. Helena," which is to be given on the 9th and 10th prox., in aid of the St. Helena Home for Nurses. The performance is to be supported by some distinguished artistes.

Sir William Smart, M.D., of West Kensington, Inspector General of Hospitals, has been awarded a good service pension of £150 a year.

Miss Kirwood has been appointed matron of the Beverley Cottage Hospital.

Miss Proctor, of the London Throat and Ear Hospital, has been appointed matron of the Middlesborough Fever Hospital. There were fifty applicants for the post.

Dr. William Graham, of the Belfast Lunatic Asylum, has been appointed Medical Superintendent to the Armagh District Lunatic Asylum.

Dr James Barr, physician to the Stanley Hospital, has been elected an honorary physician to the Northern Hospital, Liverpool, in the room of Dr. Caton, resigned.

Mr. Brand Sapte, C.B., of 116, Gloucester Terrace, W., has generously presented the nurses at the Paddington Infirmary with a piano for their Recreation Room. As the nurses' room is in the Administrative Block, and separated from the wards by a corridor, the nurses will be able to make use of Mr. Sapte's handsome present without disturbing the patients.

Dr. Dunlop D. Costine and Mr. E. Mason Sheldon, M.R.C.S., the founders of the Stanley Hospital, Liverpool, have been presented by the medical staff, with portraits in oils, in recognition of their valuable services during the past twenty years as honorary physician and honorary surgeon of the institution.

No less than 4,668 patients were treated last year at the Eastern Dispensary, Bath, the largest number recorded for the past twenty-three years. In 1853 there was a slightly higher total, but then the Southern and Western Dispensaries had not been established.

The report of the Bristol Dispensary for the past year is most satisfactory. In the midwifery department, which is a special feature of this charity, not a single death occurred out of 472 cases attended. It is expected that the new premises will be finished by the end of the year.

The Convalescent Home for Poor Children at Hastings admitted last year 846 children, of whom no less than 592 came from London and the suburbs. The Princess of Wales has recently become a life member of the Home.

At a recent meeting at the Canterbury Guildhall a resolution was passed in favour of endowing a Victoria Ward at the Kent and Canterbury Hospital, commemorative of the Jubilee.

The Governors of the Wrexham Infirmary are making a special appeal for increased support. The average income of the institution is about £800, while the expenditure exceeds this figure by £150.

The drainage of the Chichester Infirmary is under going complete reconstruction. Some unnecessary alarm was recently created, and the committee had the Infirmary closed, about thirty patients being sent to their homes. The institution has, however, been reopened, and the work will not, it is thought, in any way endanger the health of the inmates.

The Building Fund of the new Provident Dispensary for Bedford now amounts to rather over £1,400.

A Cottage Hospital has been proposed for Welshpool as a suitable local commemoration of the Queen's Jubilee. At present the town is merely provided with a Dispensary, sufferers requiring hospital treatment now having to go to Oswestry, Newtown, or Shrewsbury.

At the Annual Meeting of the Governors of the Canterbury Dispensary, held on Thursday, the 6th inst., the congratulations of the Committee were offered to the friends of the institution upon the completion of the Jubilee Year. The annual report was read, and stated that since the opening of the dispensary in Jan., 1837, the large number of 76,210 persons had received attention, and nearly 90 per cent. of this total had had the benefit of medical aid in their own homes. The number of patients during the last year was 1,869. The report concluded with a recommendation that, "in consideration of the fifty years' service of Mr. Rigden, your medical officer, a gratuity of £50 be presented to him by the Governors."

THE following vacancies are announced this week, the amount of the salary being placed in each case after the name of the institution :—

**Honorary Medical Officers.**

Hospital for Epilepsy, etc., Regent's Park. Assistant Physician.

**Resident Medical Officers.**

Brompton Consumption Hospital. Several Clinical Assistants.
Carlisle County Asylum. Junior Medical Assistant.—£80, with board.
Great Northern Central Hospital. Two Clinical Assistants.
Hull Dispensary House Surgeon.—£150, and house.
Lancashire County Asylum. Assistant.—£100, with board.
Middlesborough Infirmary. House Surgeon, doubly qualified. £100, with board.
Reading Hospital. Assistant House Surgeon. Board, no salary.
Shrewsbury Eye Hospital. F.R.C.S.E. or M.R.C.S.E.—£150.

**Non-Resident Medical Officers.**

National Orthopœdic Hospital. Surgical Registrar and Anæsthetist. Registered.—£20.

**Trained Nurses.**

Bedford Infirmary. One Night.—£22. One Assistant.—£20.
Coventry Nursing Association. One.—£30, with board.
Cambridge Nurses' Home. Two for private nursing.
Paddington Workhouse Infirmary. Two.—£20, with board, etc.
Suffolk General Hospital. Surgical nurse.—£25, with uniform, etc.

**Probationers.**

Glasgow Maternity Hospital. Several. Fee £10.
Isle of Man Hospital One.

THE erection of the Great Northern Central Hospital can, unfortunately, proceed only piece-meal. One block, which will provide sixty beds, has been put in hand, the total cost of which will be £20,000. This is all that the Committee feel justified at present in attempting. The out-patients' accommodation must, for a time at least, remain in its present unsatisfactory state. At the half-yearly meeting of the General Council last week, the chairman, Mr. D. Marshall Lang, threw out a very good suggestion, viz., that as the out-patient department is a branch which mainly concerns the working-men of the neighbourhood, they should make its erection their own special contribution. It is proposed that the foundation stone shall be laid early in the spring, and efforts are being used to secure the presence of a member of the Royal Family on that occasion.

THE Kensington District Nursing Association, the work of which we recently noticed, is making very efficient progress. It has been found necessary to add another nurse to the three already employed. From its start in April 1885 to 1st December last, the nurses of the Kensington Branch of the District Nursing Association attended over 400 cases and paid 8,000 visits. The annual cost of maintaining the four nurses amounts to about £550.

FROM the far West of England comes a proposal—and a very excellent one, too—on behalf of poor mine women who labour with sledge, with shovel, and with wheelbarrow at a wage, frequently, of only a shilling a day. These daughters of toil, who perform duties so arduous and so foreign to their sex, are a quiet uncomplaining class, who labour on, exposed to all weathers of our "brumous Albion," till the grave relieves them of their hard lot. Some years ago, the late Lord Robartes established a Miners' Hospital, an institution which the present Lord and other friends of the mining community (including Mr. and Mrs. Basset of Tahidy) have nobly supported. Dr. Permewan, who has a wide acquaintance with the miners of Cornwall, is endeavouring to establish for the mine women a small home or hospital of a similar character, and there are some premises in Redruth which, he thinks, might be secured for the purpose. Such an institution, Dr. Permewan believes, might be supported at a cost of about £400 a year. Here is an opportunity which the rich land-owners of Cornwall might worthily seize. A Jubilee Hospital for the mine women would be one of the most fitting memorials which the county could produce, to say nothing of the incalculable boon which it would prove to the poor women whose toil brings so large a return to the wealthy owners of the land.

THE Local Government Board have written to the Paddington Guardians stating that they will defer their decision upon the scheme for the appointment of clinical assistants at the Infirmary, until the control of the workhouse medical work is vested in the Medical Superintendent of the Infirmary. At present the post of Workhouse Medical Officer, and Medical Superintendent of the Infirmary, are held by different gentlemen.

IT is earnestly to be hoped that the new hospital, which will be opened during the present year at Hastings, will start upon its errand of mercy free of debt. The bazaar which the ladies of Hastings and St. Leonard's recently organised with so much success, realised over £1,700, but there still exists a debt of £5,500. There is some talk that the hospital may not unlikely be opened by royalty.

THE Bournemouth Jubilee Hospital Fund is making very rapid progress, although no serious canvass has yet been made to bring in subscriptions. Amongst the donations promised or given are : £250 from Mr. G. J. Fenwick ; £200, Miss Durrant ; £105, Dr. J. Roberts Thomson ; £105, Captain Hartley ; and £100 each from Mr. G. W. Petter, Miss M. Wilken, and Mr. H. Munro. Altogether about £1,550 has been subscribed.

IF the annual ball in aid of the Stockport Infirmary receives a measure of patronage next year similar to that accorded to it on Wednesday week, the stewards will be wise in finding a larger ball room than the Pendlebury Memorial Hall. The company numbered about 250, including both the borough members, Mr. Jennings and Mr. Gedge. Lieut.-Colonel Staples was assiduous in making the arrangements, and the success of the ball was largely due to his thought and care.

MORE hospitals for London ! It is devoutly to be hoped that we shall *not* have such an infliction. The proposed Jubilee Hospital at Gloucester Terrace, Queen's Gate, is to deal with diseases of the ear, throat, skin, eye, etc. A reference to our "Hospital Diary" will show that London is not ill-provided with special hospitals for all these cases, and it certainly seems to us the height of the folly, when so many charities are on the verge of bankruptcy, to multiply their number.

THE following Hospital Sunday fixtures have been made: February 13th, Shrewsbury; March 13th, Southampton; October 9th, Nottingham.

THE following sums have been recently received by the various Medical Charities, the name of the donor or the source from which they were obtained being given in each instance after the name of the Institution:—

**Legacies.**

Glasgow Royal Infirmary. Mr. Thomas Lockerley. £100.
Leeds Infirmary. Mr. William Neal. £200.
Addenbrooke's Hospital, Cambridge. Mr. R. L. Clark. £100.
St. Mary's Hospital, Paddington. Mrs. A. M. Gaselee. £100.
Grantham Hospital, Grantham. Miss Bestwick. £50.
Grimsby Hospital. Mr. Wood. £200.
Norfolk and Norwich Hospital. Mrs. Harriet Hayward. £500.
York County Hospital. Mr. J. Skelton. £500.

**Proceeds of Entertainments.**

Blackburn Infirmary. Public Ball. £162 8*s.* 6*d.*
Ayr County Hospital. Public Ball. £400.
Uckfield Cottage Hospital. Chrysanthemum Show. £21.
Princess Alice Memorial Hospital, Eastbourne. Dramatic Entertainment at the Devonshire Park Theatre. £32 2*s.* 6*d.*
Richmond Hospital. Bazaar. £136 2*s.* 11*d.*

**Collections.**

Chester Infirmary, Hospital Saturday. £126.

**Donations.**

Taffray Hospital. Anonymous. £1,000.
Princess Alice Memorial Hospital, Eastbourne. Mrs. Kettlewell, £50. Mr. G. Gurney, £50.
Aberdeen Royal Infirmary. The Lord Provost Henderson, £1,000.
British Home for Incurables. "A Friend", 100 Guineas.
West London Hospital, Hammersmith. Mrs. Hull Martin, £100.
Bristol Eye Hospital. Lady Cave, £25; Mrs. M. Seymour, £10: Messrs. Sheldon, Bush, and Co., £10.
City of Dublin Hospital. Mrs. Hugh Browne, £10.
Kilmarnock Infirmary. Misses Wallace, £200.

ON Saturday evening the fourth of the series of entertainments to the in-patients of the Markham Square Branch of the St. John's Hospital for Diseases of the Skin took place. The concert was arranged by the "Print" Total Abstainers' Union under the directorship of Mr. E. C. Roper, the secretary. The programme displayed a most pleasing variety of song and music, and appeared to be heartily enjoyed by those of the patients who were well enough to attend. Dr. Dow gives the next entertainment on February 5th.

THOSE who know the Rev. Benjamin Waugh need not our assertion that he is the Child's Friend. We would that there were ten thousand such defenders of the helpless. No man living is, perhaps, better acquainted with the amount of child-torture which goes on in this, our Christian England, and certainly no man has done more to drag the wrong doers before the even hand of justice. The London Society for the Prevention of Cruelty to Children, Harpur Street, W.C., have issued, under the editorship of its honorary secretary, a little monthly journal, entitled *The Child's Guardian*, the great object of which is self-explained by its name. The first number is a very excellent one, and callous indeed must be the nature of the man who, on laying it down, will not feel inclined to say that the London Society for the Prevention of Cruelty to Children is one of the noblest associations existing in our midst.

AT a recent meeting of the Midland Counties Home for Incurables, held at the Arboretum, Leamington, and presided over by Lord Leigh, it was decided in future to elect patients by the votes of the subscribers, instead of by the managing committee, as hitherto. The institution is doing very satisfactorily; the income has been well kept up, and the endowment fund has been increased to about £3,000.

THE ball in aid of the little Italian Hospital in Queen Square, Bloomsbury, was successful almost beyond anticipation. The Ospedale Italiano, which we have previously had occasion to mention, was founded some three years ago by the Chevalier G. B. Ortelli, who gave the site, the house, and the furniture. The hospital was given by the Chevalier for the benefit of the poor Italian community in London, but its doors are practically open to all comers—Swiss, English, French, Swedes, etc. It has at present only twenty-four beds, and last year these were tenanted by 160 patients, while over 2,700 poor people were treated in the out-patient ward. Signor G. Ferrari, the honorary secretary, and Signor C. Bonacina, the president of the committee of organisation, were responsible for the arrangements of the ball, and they are to be congratulated upon the success which has attended their efforts. The handsome ball-room at the Holborn Town Hall was thronged by a company of nearly 500.

EVEN the photographic art may be pressed into the service of the hospital. Frequently the hours hang heavily on the convalescent patient. Anything which can be made to break this monotony is useful. Photographic views of interesting places, and likenesses of celebrities will interest a sick man, and, by causing him to think less of his illness, will tend to promote his recovery. We are glad, therefore, to see that the *Amateur Photographer* has determined to invite its readers to send in for distribution among the hospitals any disused or partially-filled albums, for which they have no special use.

MAJOR-GENERAL STOKES, the Chairman of the London Committee of the Fund for the erection of a British Hospital at Port Said, is appealing for help in this worthy undertaking. Port Said stands on the entrance to the Suez Canal, and when we remember how large a share of the traffic through the Red Sea is our own, we can readily see that a hospital for the relief of sick seamen is an institution of the utmost utility and importance. The materials for the Seamen's Hospital, are now on their way to Port Said. When they are erected, the little hospital will offer accommodation for some thirty men, and will include a dispensary, bath-room, and laundry, together with quarters for a doctor and three nurses. About £2,000 has been received for the Port Said Hospital, but Major-General Stokes estimates that about as much again is needed to furnish the hospital with stores, instruments, etc.

A HOME for Nurses is about to be built in connection with the Royal Infirmary, Glasgow. At present the nurses reside at the Institution. When the home is built, the present nurses' quarters will afford space for about sixty more beds at the Infirmary.

