# The Richmond Hospital: A Correction

**Published:** 1887-02-12

**Authors:** George C. Rowlands

**Affiliations:** Richmond Hospital, Surrey


### THE RICHMOND HOSPITAL: A CORRECTION.

Referring to your paragraph at page 302 of The Hospital, a copy of which you have been good enough to send me, allow me to point out that we have not had a bazaar for several years, and have not received the £136 2*s.* 11*d.* to which you allude. The hospital referred to should be described as St. John's Hospital, Twickenham. In case you like hospital news, I beg to enclose you a copy of my Collecting Box List for last year. The expense of collection was:—

| | |
|---|---|
| New Boxes, repairs ................ | £10 8 0 |
| Collectors' commission paid........ | 12 12 0 |
| | £23 0 0 |

thus leaving a credit balance of £257 3*s.* 6*d.* I also enclose you statistics of our work last year. The other day I received from the executors of the late Miss Maria Brown a legacy of £200.

George C. Rowlands.

Richmond Hospital, Surrey.

*** The Chairman of the Hospital has also kindly written to the same effect.

